# Annotations

**Published:** 1899-07-15

**Authors:** 


### ANNOTATIONS.

### The Air Space Question.

We regret to see a medical contemporary, from which the public naturally expects to receive reliable guidance upon matters of public health, taking up a position in regard to the housing of the working classes which is expressive rather of expediency than of the teachings of modern sanitary science. The London County Council is proposing to erect certain dwellings for the working classes, and the Local Government Board decline to pass the plans except on the condition that "the occupancy of the dwellings shall, as regards one-roomed tenements, be restricted to a childless married couple, two girls, or two elderly persons of the same sex, and as regards two-roomed tenements to two adults (being a married couple), and two children under ten years of age." On this the *British Medical Journal* comments as follows: "The Board is doubtless influenced by sound policy in endeavouring to secure a high standard of comfort and air space; but in view of the appalling overcrowded state existent in South and East and Central London, it is desirable that no red-tape officialism should fetter the well-meaning efforts of local authorities." We completely fail to see that the existence of a degree of overcrowding which is admitted to be appalling is any reason for opposing the attempts made by the Local Government Board to prevent the buildings now about to be put up from themselves in turn becoming centres of overcrowding. The opinion of a professional journal is chiefly valuable on a professional question; and if the *British Medical Journal* had contented itself with giving an opinion upon the purely medical side of the question, which was one of air space and of the healthiness or otherwise of tenements occupied by two or more persons to a room, then we feel sure that its verdict would have borne weight, and would have done much good. We venture, however, to opine that, if it had done so, it would not have described the "sound policy" of the Local Government Board "in endeavouring to secure a high standard of comfort and air space" as "red-tape officialism."

### Painful Experiments on School Children.

A question has lately arisen in America which is pretty sure to be made the text of many sermons, namely—How far may the excuse of making psychological investigations be held to warrant experiments upon children? Many investigations have been made in recent years with the object of discovering the times at which, and the conditions under which the youthful mind is the most active, and as to the sort and degree of strain which leads most certainly to mental fatigue and its accompaniments—inattention and bad work; but lately it appears that attempts have been made to measure the activity and what may be properly termed the irritability of the nervous system among different classes of children by the tendency they show to translate common sensation into pain. This seems to us to place the matter in a somewhat different category. By applying to the temple of the child under investigation an instrument which is, in fact, a pressure gauge, and ascertaining at what point the sensation aroused becomes disagreeable—much as one might pick up a puppy by the ears to see if it is "game"—it is found that the "threshold of pain" is passed by different children at very varying pressures, and by classifying the observations some not uninteresting results have been obtained; interesting, that is, to the psychologist, and to all who have to do with the training of youth in different classes of society. Thus it is found that girls in private schools, the daughters, mostly, of wealthy parents, are much more sensitive to pain than girls in the public schools; that University women are more sensitive than washerwomen, but less sensitive than business women; and that the girls in the public schools are more sensitive at all ages than the boys. All this is very interesting, although a good deal of it might have been guessed beforehand. But among civilised races the "threshold of pain" is apt to be regarded as a serious portal, notwithstanding the readiness with which it is passed by every boy in every great public school, and we fancy that in experimenting on children most people will draw the line before pain is reached. All this which has been done in America has been done under the sanction of a public department, and thus is an admirable stick with which to thrash the Government—a point worth remembering. In all seriousness, however, we think that making the children tread on the threshold of pain is a matter that should not be passed over. If a schoolmistress cannot whip a child for its own good, and a physician cannot inoculate a guinea-pig to find out what is the matter with his patient, it can scarcely be right to give the "psychologist" a free hand, and allow him to experiment on children in regard to the pain point.

### Licking the Fingers.

A tale is being told of a Parisian gentleman who refused to let the railway ticket inspector touch his ticket because his hands were dirty, being unwilling to put into his pocket a card which might be contaminated with microbes and might very likely give him some disease. After this the demands made by those who ask for separate communion cups in church, and "New" Testaments in court, fade into nothingness. The Parisian was fined a franc, which was not very ruinous, but at least it was enough to maintain the majesty of the law, as represented by the dirty-handed ticket collector. Of course, it is easy to carry one's nicety to such an extreme as to throw ridicule upon the cult of cleanliness; still, we may confess to a certain loathing of some of the little practices which are common in daily life, even in regard to tickets, and one of these habits with which we quarrel more particularly is that of temporarily increasing the adhesiveness of the finger tips by the application of saliva. The mouth is, no doubt, a very portable and convenient finger damper, but that is hardly its proper or most appropriate use, and when we find—as we often do—that an omnibus conductor, for the purpose of extracting his ticket from its bundle, moistens his finger so plentifully that the card is handed to the passenger limp and wet, we cannot but feel that the salivary secretion is being misused. It is an evil habit, one end of which is to be seen when people turn over papers and the leaves of books, and the other—well, we do not know where it ends; but we have seen a waiter lick his fingers as a preparation for slicing a tomato, and we need not go farther than that in illustration of its nastiness.

